# Single nucleotide polymorphisms (SNPs) in coding regions of canine dopamine- and serotonin-related genes

**DOI:** 10.1186/1471-2156-9-10

**Published:** 2008-01-28

**Authors:** Jørn Våge, Frode Lingaas

**Affiliations:** 1 Division of Genetics, Department of Basic Sciences and Aquatic Medicine, Norwegian School of Veterinary Science, Oslo, Norway

## Abstract

**Background:**

Polymorphism in genes of regulating enzymes, transporters and receptors of the neurotransmitters of the central nervous system have been associated with altered behaviour, and single nucleotide polymorphisms (SNPs) represent the most frequent type of genetic variation. The serotonin and dopamine signalling systems have a central influence on different behavioural phenotypes, both of invertebrates and vertebrates, and this study was undertaken in order to explore genetic variation that may be associated with variation in behaviour.

**Results:**

Single nucleotide polymorphisms in canine genes related to behaviour were identified by individually sequencing eight dogs (*Canis familiaris*) of different breeds. Eighteen genes from the dopamine and the serotonin systems were screened, revealing 34 SNPs distributed in 14 of the 18 selected genes. A total of 24,895 bp coding sequence was sequenced yielding an average frequency of one SNP per 732 bp (1/732). A total of 11 non-synonymous SNPs (nsSNPs), which may be involved in alteration of protein function, were detected. Of these 11 nsSNPs, six resulted in a substitution of amino acid residue with concomitant change in structural parameters.

**Conclusion:**

We have identified a number of coding SNPs in behaviour-related genes, several of which change the amino acids of the proteins. Some of the canine SNPs exist in codons that are evolutionary conserved between five compared species, and predictions indicate that they may have a functional effect on the protein. The reported coding SNP frequency of the studied genes falls within the range of SNP frequencies reported earlier in the dog and other mammalian species. Novel SNPs are presented and the results show a significant genetic variation in expressed sequences in this group of genes. The results can contribute to an improved understanding of the genetics of behaviour.

## Background

Neurotransmitters of the central nervous system (CNS) indisputably are important for modulation of the dispersed behaviour seen in both man and animals. Polymorphism in genes of regulating enzymes, transporters and receptors have been associated with altered behaviour [1-3]. Single nucleotide polymorphisms (SNPs) represent the most frequent type of genetic variation in human populations. Non-synonymous SNPs (nsSNPs) comprise a group of SNPs that, together with SNPs in regulatory regions, are believed to have the highest impact on phenotype [4].

The genetic basis of behaviour has been explored within a wide range of genes representing neurotransmitters and signalling molecules, and the vast majority of work has been performed on monoamine systems. The serotonin and dopamine signalling systems are central to different behavioural phenotypes, both of invertebrates and vertebrates [5-9]. Marino et al. [10] reported that defects in the noradrenergic system have been implicated in many mood, cognitive and neurological disorders that manifest abnormal social behaviour, and demonstrate that *dopamine β-hydroxylase *knock-out (*Dbh*-/-) mice are deficient in social discrimination and lack isolation-induced aggression. Monoamine oxidase (MAO) A and B play an important role in regulating levels of biogenic amines. Whereas MAOA preferentially oxidises the biogenic amines serotonin, norepinephrine and epinephrine, MAOB preferentially oxidises phenylethylamine and benzylamine. Dopamine, tyramine and tryptamine are common substrates for both forms [11]. *MAO A/B *double knock-out mice showed increased brain levels of several biogenic amines, and chase/escape and anxiety-like behaviour, suggesting that alterations of monoamine levels are implicated in a unique biochemical and behavioural phenotype [12]. Polymorphism within receptor genes of dopamine and serotonin are associated with a variety of human psychiatric disorders [13-16], and knockout models of *5HTR1B *produce a deviant mice behaviour [17].

The knowledge of genes associated to behavioural traits is increasing. Characterisations of these genes and identification of closely linked SNPs and microsatellites make it possible to study the segregation of behaviour-associated haplotypes and to learn more about the genetic contribution to canine behaviour. SNPs as abundant polymorphisms scattered over the genomes are important tools for detailed mapping [18]. Beside their value as markers, some of these variations represent polymorphisms with functional effects. Descriptions of genetic variation in expressed sequences and changes in protein sequence may contribute to reveal the causes of differences in behavioural phenotypes.

The large number of canine breeds exhibits an extreme between-breed variation in traits like size, colour, conformation and behaviour. For many of these breeds, behavioural characteristics represent an important part of the breed definition and description. Certain behavioural phenotypes are associated with specific breeds as a result of long-term, systematic selection and limited genetic variation. In a behavioural context, dog breeds are evidence for the considerable impact of genetics on behavioural traits. They are therefore valuable models for genetic studies aimed at revealing basic biological knowledge of genetic regulation of behavioural traits. This can be efficiently performed through crossbreeding and backcrosses of these isolates with strong between-breed contrasts in specific behaviours.

Some recent publications characterise polymorphisms in the canine dopamine and serotonin gene families [19-23], but the number of reported SNPs in coding sequence of behaviour-related genes from dogs is still low. A better knowledge of genetic variation in these genes will be important for an improved understanding of the genetic influence on behaviour in both animals and humans. This study presents novel SNPs in coding sequences of canine serotonin- and dopamine-related genes.

## Results

Sequencing a total number of 24,895 bp coding DNA in each of eight dogs of different breeds revealed a total of 34 SNPs, 30 of them not earlier reported, distributed in 14 of the 18 selected genes (Table 1). SNPs were identified in five genes in the dopamine pathway, in one gene related to synthesis of norepinephrine and in nine genes in the serotonin pathway (Table 2).

**Table 1:** List of genes included in the study, with sequence screened and SNP frequency


**Gene**	**Gene symbol**	Gene product category/function*	**Bp screened**	**SNP/bp**

Dopamine related genes				
*Tyrosine hydroxylase*	*TH*	Presynaptic form. of DOPA	1488	1/1488
*Dopamine transporter*	*SLC6A3*	Synaptic clearance of Dopamine	1881	1/470
*Dopamine receptor D1*	*DRD1*	G protein-coupled receptor	1341	1/335
*Dopamine receptor D2*	*DRD2*	G protein-coupled receptor	1332	1/666
*Dopamine receptor D3*	*DRD3*	G protein-coupled receptor	1344	1/336
				
Norepinephrine related gene				
*Dopamine beta hydroxylase*	*DBH*	Presynaptic form. of norepinephrine	1839	1/306
				
Serotonin related genes				
*5-hydroxytryptamine receptor 1A*	*HTR1A*	G protein-coupled receptor	1272	1/1272
*5-hydroxytryptamine receptor 1B*	*HTR1B*	G protein-coupled receptor	1170	1/585
*5-hydroxytryptamine receptor 1D*	*HTR1D*	G protein-coupled receptor	1134	1/283
*5-hydroxytryptamine receptor 1E*	*HTR1E*	G protein-coupled receptor	1098	1/1098
*5-hydroxytryptamine receptor 1F*	*HTR1F*	G protein-coupled receptor	1101	1/1101
*5-hydroxytryptamine receptor 2A*	*HTR2A*	G protein-coupled receptor	1414	0
*5-hydroxytryptamine receptor 2B*	*HTR2B*	G protein-coupled receptor	1305	1/652
*5-hydroxytryptamine receptor 2C*	*HTR2C*	G protein-coupled receptor	1377	1/1377
*5-hydroxytryptamine receptor 3A*	*HTR3A*	Ligand-gated ion channel	1452	1/1452
*5-hydroxytryptamine receptor 4*	*HTR4*	G protein-coupled receptor	1167	0
*Serotonin transporter*	*SLC6A4*	Synaptic clearance of Serotonin	1845	0
*Tryptophan hydroxylase 1*	*TPH1*	Presynaptic form. of hydroxytryptophan (5HT)	1335	0

Sum of bp screened			24895	

No. of SNPs detected			34	
Frequency of SNPs (snp/bp)			1/732	
Synonym (n = 23)			1/1082	
Non-synonym (n = 11)			1/2263	

* As defined at [35]

**Table 2:** SNPs according to position, function, predicted changes in amino acid and novelty.


**Gene**	CFA*	**Region**	**Allel**	**Function^†^**	**Codon position**	**Protein residue^‡^**	**Aa position**	**Novelty of SNP^§^**	**Reference sequence^||^**

*TH*	18	EX 13	C/T	syn	3	Asp [D]	482	Novel	[GenBank: NM_001002966.1]
*SLC6A3*	34	EX 9	A/G	syn	3	Gly [G]	418	Novel	[GenBank: XM_846543.1]
		EX 10	C/T	syn	3	Asp [D]	468	Publ. – rs23877306	[GenBank: XM_846543.1]
		EX 10	A/G	syn	3	Ala [A]	471	Novel	[GenBank: XM_846543.1]
		EX 11	C/T	syn	3	Asp [D]	498	Novel	[GenBank: XM_846543.1]
*DRD1*	4	EX 1	A/G	syn	3	Ala [A]	84	Novel	[GenBank: XM_546227.2]
		EX 1	C/T	syn	3	Gly [G]	88	Novel	[GenBank: XM_546227.2]
		EX 1	C/T	syn	3	Val [V]	236	Novel	[GenBank: XM_546227.2]
		EX 1	C/T	ns	2	Thr [T]/Met [M]	354	Novel	[GenBank: XM_546227.2]
*DRD2*	5	EX 2	C/T	syn	3	Asn [N]	23	Novel	[GenBank: NM_001003110.1]
		EX 2	C/T	syn	3	Ala [A]	77	Novel	[GenBank: NM_001003110.1]
*DRD3*	33	EX 4	C/T	syn	3	Cys [C]	231	Novel	[GenBank: XM_545106.2]
		EX 6	A/G	ns	2	Gln [Q]/Arg [R]	294	Novel	[GenBank: XM_545106.2]
		EX 6	C/T	ns	1	Leu [L]/Phe [F]	341	Novel	[GenBank: XM_545106.2]
		EX 7	C/T	syn	3	Cys [C]	402	Novel	[GenBank: XM_545106.2]
*DBH*	9	EX 1	A/G	syn	3	Thr [T]	17	Novel	[GenBank: NM_001005263.1]
		EX 2	A/G	syn	3	Gln [Q]	121	Novel	[GenBank: NM_001005263.1]
		EX 3	C/T	syn	3	Tyr [Y]	163	Novel	[GenBank: NM_001005263.1]
		EX 4	A/C	ns	3	Lys [K]/Asn [N]	263	Novel	[GenBank: NM_001005263.1]
		EX 4	C/T	syn	3	Gly [G]	297	Novel	[GenBank: NM_001005263.1]
		EX 12	A/G	syn	3	Gly [G]	622	Novel	[GenBank: NM_001005263.1]
*HTR1A*	2	EX1	A/C	ns	1	Lys [K]/Gln [Q]	270	Publ. – rs22855024	[GenBank: XM_544358.1]
*HTR1B*	12	EX 1	A/C	ns	1	Ile [I]/Leu [L]	53	Novel	[GenBank: NM_001006948.1]
		EX 1	A/G	syn	3	Pro [P]	82	Novel	[GenBank: NM_001006948.1]
*HTR1D*	2	EX 1	C/T	ns	2	Ala [A]/Val [V]	97	Novel	[GenBank: NM_001003280.1]
		EX 1	C/T	syn	3	Tyr [Y]	221	Novel	[GenBank: NM_001003280.1]
		EX 1	A/G	ns	2	Glu [E]/Gly [G]	263	Publ. – rs22791523	[GenBank: NM_001003280.1]
		EX 1	C/T	syn	3	His [H]	372	Novel	[GenBank: NM_001003280.1]
*HTR1E*	12	EX 1	C/T	syn	1	Leu [L]	39	Novel	[GenBank: XM_539028.1]
*HTR1F*	31	EX 1	C/T	syn	3	Arg [R]	162	Publ. – rs9250875	[Ensembl: ENSCAFT00000012417]
*HTR2B*	25	EX 1	C/T	ns	2	Thr [T]/Ile [I]	88	Novel	[GenBank: NM_001024633.1]
		EX 3	C/T	ns	2	Ala [A]/Val [V]	431	Novel	[GenBank: NM_001024633.1]
*HTR2C*	X	EX 6	A/G	syn	3	Pro [P]	280	Novel	[GenBank: NM_001006648.1]
*HTR3A*	5	EX 2	C/T	ns	2	Thr [T]/Met [M]	64	Novel	[GenBank: XM_546517.2]

* Chromosome number, *Canis familiaris*.

† Synonymous SNP (syn) and non-synonymous SNP (ns)

‡ Listed ambiguity is decided by the respective allel in column 4.

§ Novelty of SNPs explored by blasting the dbSNP (Dog_9615) at [36]. Reference no. given for corresponding published SNP.

|| Canine sequence/predicted sequence, for defining SNPs position in protein. Given as GenBank or Ensemble accession number.

**Figure 1:**
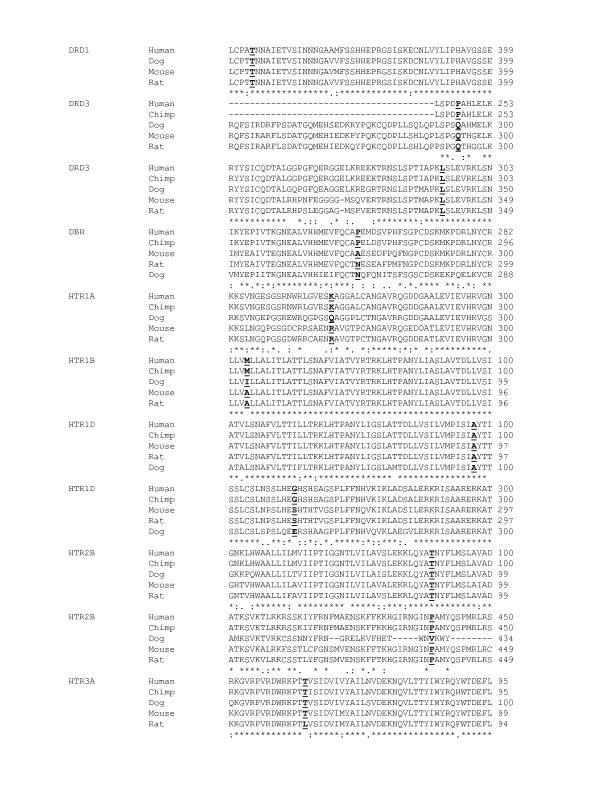
**Alignment showing amino acid variation across five species in location of nsSNPs**. The alignment show selected parts of the protein sequence containing the 11 detected nsSNPs in the dog, marked as bold and underlined residues. Only four species are aligned for the DRD1 gene since there was no homologous gene available for the Chimpanzee (HomoloGene, [24]).

The 34 SNPs comprised 23 synonymous and 11 non-synonymous, with the predicted changes in amino acids as described in Table 2 (for flanking nucleotide sequences see Additional file 1). Of the 11 nsSNPs, three held the first position, seven held the second position and one held the third position of the codon. Categorisation of the SNPs according to nucleotide substitution gave 31(91%) transitions and 3 (9%) transversions, the transversions all being nsSNPs. Six of the 11 nsSNPs resulted in a substitution of amino acid residue with a concomitant shift of class dependent on R group, and change in structural parameters (Table 3). Looking at conservation of amino acids in the location of detected nsSNPs we found that across five mammalian species (*Homo sapiens, Pan troglodytes, Canis familiaris, Mus musculus *and *Rattus norvegius*, at HomoloGene, [24]) four of the sites were reported invariant and seven reported variable (Table 3). Part of the alignment of the protein products from these five species, containing the canine nsSNPs (ClustalW, [25]) are shown in Figure 1.

**Table 3:** Shift of residues with change of class according to R groups, conservation across species and PolyPhen predictions.


**Gene**	Protein residue*	**Aa position**	**Change in class according to R group^†^**	**Residue conservation across 5 species^‡^**	**PolyPhen prediction^§^**

*DRD1*	Thr [T]/Met [M]	354	No change	Invariant	Possibly damaging
*DRD3*	Gln [Q]/Arg [R]	294	Polar, uncharged/positively charged	Variable	Benign
	Leu [L]/Phe [F]	341	Nonpolar, aliphatic/aromatic	Invariant	Benign
*DBH*	Lys [K]/Asn [N]	263	Positively charged/polar, uncharged	Variable	Benign
*HTR1A*	Lys [K]/Gln [Q]	270	Positively charged/polar, uncharged	Variable	Benign
*HTR1B*	Ile [I]/Leu [L]	53	No change	Variable	Benign
*HTR1D*	Ala [A]/Val [V]	97	No change	Invariant	Benign
	Glu [E]/Gly [G]	263	Negatively charged/Nonpolar, aliphatic	Variable	Benign
*HTR2B*	Thr [T]/Ile [I]	88	Polar, uncharged/nonpolar, aliphatic	Invariant	Probably damaging
	Ala [A]/Val [V]	431	No change	Variable	Predicted to be unknown
*HTR3A*	Thr [T]/Met [M]	64	No change	Variable	Possibly damaging

* Changes according to Table 2, shift in residues as result of different alleles of SNPs.

† Changes according to shift of residues in column 2, respectively. Classes according to R groups as described by [37].

‡ Residue variation across five mammalian species.

§ Prediction of a possible damaging effect of the amino acid substitutions caused by the nsSNPs, performed with PolyPhen [4].

The potential functional effects of the 11 identified substitutions caused by the nsSNPs were explored using the software PolyPhen [4], designed to predict functional effects of amino acid substitutions (in humans). The predictions are classified as unknown, benign, possibly damaging and probably damaging. The results showed that the effect of the amino acid substitution was predicted to change the function in three of the residues (possibly/probably damaging) and was classified as benign in seven of the substitutions. In one of the substitutions the effect was unknown (Table 3).

## Discussion

Being the most frequent variation of DNA, SNPs represent important causes of transcript variation. The identification and closer study of these polymorphisms are important for the assignment of the genetic contribution to different phenotypes. This study describes SNPs in genes from neurotransmitter systems that are reported to be related to different behavioural phenotypes.

SNP frequencies show a considerable variation between species [18,26-28], and Lindblad-Toh et al. [29] presents in dog a between-breed SNP frequency of ~1/900 bp based on shotgun sequence data from each of nine diverse breeds compared to the boxer genome. The SNP frequency detected in our study (1/732, see Table 1), where a higher number of chromosomes are compared, falls within this range. We are not aware of prior studies reporting SNP frequency of coding sequences from a number of canine genes.

In our study we observed a ~2.5 times higher number of SNPs in dopamine-related genes compared to serotonin-related genes. In the group of dopamine-related genes we observed 15 SNPs/7,386 bp sequenced (1/492), while 12 SNPs/14,218 bp (1/1184) were observed in the group of serotonin-related genes (p < 0.05). This may indicate a greater conservation or a greater similarity in the gene structure of the serotonin-related genes compared to the group of dopamine-related genes. The two gene sets represent genes with similar function related to the respective neurotransmitters. The G-coupled receptors are, however, more numerously represented among the serotonin related genes (Table 1). One gene, HTR3A is a ligand-gated ion channel and is kept out of the analysis.

Since the completion of the sequence for several genomes, there has been an increased focus on functional polymorphism. Databases containing huge numbers of SNPs are now available for the research community. Besides outlining genome architecture with gene location and description of polymorphisms, one of the major challenges is to infer the functional implications of these variations. It has been estimated that ~20% of common human nsSNPs damage the protein [30]. A large database for identification of human nsSNPs with potential impact on disease (PolyDoms, [31]) uses two sequence homology-based tools, SIFT [32] and PolyPhen [4], to predict the potential impact of nsSNP on protein function. Among the structural parameters analysed in PolyPhen for assessing a possible damaging effect of amino acid substitutions are properties in relation to changes of hydrophobicity and electrostatic charge, as well as protein solubility and compatibility of amino acid substitutions in homologous proteins. The changes of R-group classes seen in six of the substitutions in our study (Table 3) represent a change in such structural parameters. When inferring about the effect of the predicted amino acid substitutions it can be useful to combine data describing biochemical properties of residues, with knowledge of the conservation across species. Table 3 shows that four residues are evolutionary conserved between the five compared species. Of these, two also experience a change in class of R-group. Presumably one would expect these two substitutions to be the ones most likely to cause functional changes in the protein

## Conclusion

We have identified a number of coding SNPs in behaviour-related genes, several of which change the amino acids of the proteins. Some of the canine SNPs exist in codons that are evolutionary conserved between five compared species, and predictions indicate that they may have a functional effect on the protein. The reported coding SNP frequency of the studied genes falls within the range of SNP frequencies reported earlier in the dog and other mammalian species. Novel SNPs are presented and the results show a significant genetic variation in expressed sequences in this group of genes. The results can contribute to an improved understanding of the genetics of behaviour.

## Methods

### Materials

Blood samples were collected from eight dogs of eight different breeds – rottweiler, Labrador retriever, Newfoundland, golden retriever, English setter, boxer, Norwegian lundehund and German shepherd. All dogs were healthy pets visiting the veterinary clinic for routine control.

### DNA isolation

DNA was isolated from 10 ml of EDTA-blood by the phenol-chloroform method [33]. DNA was aliquoted and stored at -20°C.

### Identification of genomic sequences

The initial identification of relevant canine sequence was performed using comparative genomics, facilitated through the high degree of similarity between human and canine genomes [28,29]. Published human and canine sequences from NCBI and ENSEMBLE were aligned and primers were designed to amplify exonic sequence (primer 3, [34]).

The selected exonic sequences originated from a total of 18 genes, consisting of nine serotonin G protein-coupled receptors and one ligand-gated ion channel, three dopamine G protein-coupled receptors and additionally exons from four genes related to serotonin and dopamine formation and synaptic clearance. The study also included one enzymatic gene related to synthesis of norepinephrine (Table 1).

### PCR amplification and SNP detection

Primers flanking each of the exon sequences of 12 serotonin-, five dopamine- and one norepinephrine-related genes (Table 1) were run in PCR with ~25 ng of canine genomic DNA as template, 1.5 μl 10× PCR buffer containing 15 mM MgCl_2 _(Qiagen), 0.6 μl dNTP (2.5 mM), 0.5 μl PCR primer, forward and reverse (5 pmol/μl), 0.05 μl Taq DNA Polymerase (5 U/μl, Qiagen) and water to a total volume of 15 μl. Initial denaturation at 95°C (2 1/2 min.), followed by 34 cycles of 95°C (30 sec.), 58°C (40 sec.), 72°C (50 sec.) and finally at 72°C (5 min.). Primers not providing specific PCR products by these conditions were run at 95°C (2 1/2 min.), followed by 34 cycles of 95°C (30 sec.), 60°C (40 sec.), 72°C (50 sec.) and finally at 72°C (5 min.).

The obtained PCR products were sequenced in both forward and reverse directions with the same PCR primers, by the MegaBACE™ 1000 DNA Analysis Systems (Amersham Biosciences) using the DYEnamic™ ET Dye Terminator Kit (Amersham Biosciences). Reaction conditions were as follows: 4 μl ET reagent premix, 4.5 μl H_2_O, 1 μl PCR-product and 0.5 μl primer (5 μM) with the following step repeated 28 times: 95°C (15 sec.), 58°C (10 sec.), 60°C (1 min.). The post-reaction cleanup was performed as recommended by the protocol with ethanol and 7.5 M ammonium acetate. SNPs were identified by aligning and comparing the sequence data with Sequencher 4.1.4 (Gene Codes Co.)

### SNP description and possible amino acid change

Reference sequences were displayed from available databases and open reading frames (ORFs) defined. Further alignment and translation with Sequencher 4.1.4 (Gene Codes Co.) defined the codons and amino acid changes (Table 2). Alignment of protein sequences with nsSNPs (reference sequences in Table 2) for detection of conservation across species was performed with ClustalW [25]. Prediction of a possible damaging effect of the amino acid substitutions caused by the nsSNPs was performed with PolyPhen [4].

## Authors' contributions

JV carried out the majority of the molecular genetic studies, performed the analysis and drafted the manuscript. FL conceived of the study, and participated in its design and coordination and helped to draft the manuscript. Both authors read and approved the final manuscript.

## Supplementary Material

Additional file 1 "Reported SNPs with flanking nucleotide sequences". SNPs, listed according to Table 2, with 200 bp flanking sequences. Click here for file
