# Supplementary material for: Single nucleotide polymorphisms (SNPs) in coding regions of canine dopamine- and serotonin-related genes
Source: BMC Genet. 2008 Jan 28;9:10. doi: 10.1186/1471-2156-9-10 (PMC2268707; doi:10.1186/1471-2156-9-10)
Supplement: Additional file 1 — "Reported SNPs with flanking nucleotide sequences". SNPs, listed according to Table 2, with 200 bp flanking sequences. [file 1471-2156-9-10-S1.pdf]

Reported SNPs with flanking nucleotide sequences

SNP local name	SNP with 200bp flank	Aa position
THsnp1	GGCTGGCCCACTCCTCCCTATCTCCCCAGTGCCCCCCGAGTCCCACCA<br>GGTGACCGAGGGCCCATATGCCCTGCCCTCCCTAGGAACTACGCTTC<br>CCGCATCCAACGCCCCTTCTCCGTGAAGTTCGACCCATATACGCTGGC<br>CATCGACGTGCTGGACAGTCCCCACGCCATCCGGCGCTCGCTGGAGG<br>GGGTCCAGGA**[C/T]**GAGCTGCACACCCTCGCCCATGCGCTCAGCGCCA<br>TCGGCTAGCTGCAGCCGGGAGAGGCCCCTGCAGTCTCCTCACCCTTC<br>CCCGGCCTCCCCCGGCCCCTGCCCAAGCAAGGGGCTGGGGCACTGG<br>GCACCCCCAAGAGCCTGCCAGAGACCCTCCCCACGGGAAGGCACCCC<br>ACCAACCCCCACCCTGCTGCTTCCTG	482
SLC6A3snp1	CTGGGGGAGGTGGTTTCGACAGAGCAGGGCAGGGAGGGTCTCCAAG<br>GAGTCCCGGGTGTTTGGATGACACCCCATCATGACGACTGCACCCCAG<br>ATCTGGCGGCCCCCACTCGTGTCCACACCCCAGGGGTAGGCCGGTTG<br>AAGTCACGTAAGAGGCGCCTGCCCCACAGCACCCCTGTCTGTTGGCT<br>GCAGATGGGTGG**[A/G]**ATGGAGTCGGTGATCACTGGGCTCATCGACGA<br>GTTCCAGCTGCTGCACAGGCACCGGGAGCTCTTCACCCTCTTCATCGT<br>CCTGGCCACCTTCCTCCTCTCGCTCTTCTGTGTCACCAACGTACGTAC<br>CCCTGCTCGCTTTGCCTGCGAGGTCTCGCTAGCGTTTGGTGTCGGGC<br>AGCGTGCTTGGTGGCTGCGGGCCAG	418
SLC6A3snp2	AGAAGGCCTGTCCCTGAGAAGCCGTGTGGCTTGGATTTCAGTGACTCA<br>GTGGGACTCCACAGGAGGAAGGTGGCCCCAGGCCCCGAGTGTTTTGG<br>GAAAGTGCCCTGCGGGCTTCTGTGGGCGTGGAGGCCACAGTGGGCAC<br>CCCACGCTCAGCGGGCTCTCCTCCTCCAGGGCGGCATCTACGTCTTCA<br>CGCTGCTGGA**[C/T]**CACTTTGCGGCCGGCACATCCATCCTCTTTGGGG<br>TGCTCATCGAGGCCATTGGGGTGGCCTGGTTCTACGGTAAGGATGGA<br>GTGCTGTCCGCGGCCGGAGAGCCTCAGCAGGTGGAGGACCCGGCCG<br>CGGTGCCCGCACAGTGAGCGGCCGGGCTGGTGGCCTCGCACTTTGTC<br>CCCGGGCTGCATCCTTCCTGACAAAG	468
SLC6A3snp3	GTCCCTGAGAAGCCGTGTGGCTTGGATTTCAGTGACTCAGTGGGACTC<br>CACAGGAGGAAGGTGGCCCCAGGCCCCGAGTGTTTTGGGAAAGTGCC<br>CTGCGGGCTTCTGTGGGCGTGGAGGCCACAGTGGGCACCCCACGCTC<br>AGCGGGCTCTCCTCCTCCAGGGCGGCATCTACGTCTTCACGCTGCTG<br>GATCACTTTGC**[A/G]**GCCGGCACATCCATCCTCTTTGGGGTGCTCATCG<br>AGGCCATTGGGGTGGCCTGGTTCTACGGTAAGGATGGAGTGCTGTCC<br>GCGGCCGGAGAGCCTCAGCAGGTGGAGGACCCGGCCGCGGTGCCCG<br>CACAGTGAGCGGCCGGGCTGGTGGCCTCGCACTTTGTCCCCGGGCTG<br>CATCCTTCCTGACAAAGCCACCATGG	471
SLC6A3snp4	GACTGAGGGCACCCCTGCAGGACATGGGGGACCCGGGGCCGAGGCT<br>GCTGGGAGGTCAGGAGTGGGGGCGCCCCCGCAGGATGTGGGGGACC<br>CGGGGCCGAGGCTGCTGGGAGGTCAGGAGTGGCACAGGCCGAGGCT<br>CGGGGTGCCGTCCCGACCGGCCGCTCCACTCCTTGCACCTCAGGCGT<br>GAGGCAGTTCAGCGA**[C/T]**GACATCAAGCAGATGACCGGGCAGCGGCC<br>CAGCCTCTACTGGAGGGTGTGCTGGAAGTTCGTCAGCCCCTGCTTCCT<br>CCTGGTATGGGCCCTGCGGCCGCCCACACTGCTAAGGCTGGGGAGG<br>GGGCTCCTGCGATCCCTGAAAATCCTCTGTGGTCTCCTGAAGGCAGCC<br>CCCACCCTCTGGACCCAGGCTCCAGGGCA	498

DRD1snp1	AGAGACTTCTCATTCCGCATCCTTACAGCCTGTTTCCTGTCTCTGCTCA<br>TCCTGTCCACACTGCTGGGGAACACACTGGTCTGTGCCGCTGTCATCA<br>GGTTCCGACACCTGCGGTCCAAGGTGACCAACTTCTTTGTCATCTCCT<br>TGGCCGTGTCAGATCTCTTGGTGGCCGTCTTGGTCATGCCCTGGAAAG<br>CGGTGGC**[A/G]**GAGATCGCTGGCTTCTGGCCCTTTGGGTCCTTCTGTA<br>ACATCTGGGTGGCCTTTGACATCATGTGCTCCACTGCGTCCATCCTCA<br>ACCTCTGTGTGATTAGCGTGGACAGGTATTGGGCCATCTCTAGTCCCT<br>TCCGGTATGAGAGGAAGATGACCCCCAAGGCAGCCTTCATTCTGATCA<br>GCGTGGCGTGGACCTTGTC	84
DRD1snp2	TTCCGCATCCTTACAGCCTGTTTCCTGTCTCTGCTCATCCTGTCCACAC<br>TGCTGGGGAACACACTGGTCTGTGCCGCTGTCATCAGGTTCCGACACC<br>TGCGGTCCAAGGTGACCAACTTCTTTGTCATCTCCTTGGCCGTGTCAG<br>ATCTCTTGGTGGCCGTCTTGGTCATGCCCTGGAAAGCGGTGGCAGAG<br>ATCGCTGG**[C/T]**TTCTGGCCCTTTGGGTCCTTCTGTAACATCTGGGTGG<br>CCTTTGACATCATGTGCTCCACTGCGTCCATCCTCAACCTCTGTGTGAT<br>TAGCGTGGACAGGTATTGGGCCATCTCTAGTCCCTTCCGGTATGAGAG<br>GAAGATGACCCCCAAGGCAGCCTTCATTCTGATCAGCGTGGCGTGGAC<br>CTTGTCTGTACTTATCTC	88
DRD1snp3	AGTTCCTCCGATGGGAATGCCACTTCCCTGGGTGAGACCATGGACAAC<br>TGTGATTCCAGCTTAAGCAGGACATATGCCATTTCATCCTCCCTAATAA<br>GCTTTTATATCCCCGTGGCCATCATGATTGTCACCTACACCAGGATCTA<br>TAGGATCGCCCAGAAACAAATACGGCGCATTTCGGCCTTAGAGAGGGC<br>AGCAGT**[C/T]**CATGCCAAGAATTGCCAGACCACTACAGGTAATGGAAAC<br>CCTGTGGAGTGTTCTCAACCAGAAAGCTCCTTTAAGATGTCCTTCAAGA<br>GAGAGACTAAAGTTCTGAAGACTCTGTCTGTGATCATGGGGGTATTTGT<br>GTGCTGCTGGCTTCCTTTCTTCATCTTGAACTGCATGGTGCCCTTCTGT<br>GGGTCTGGGGAGAC	236
DRD1snp4	TTTCTTCATCTTGAACTGCATGGTGCCCTTCTGTGGGTCTGGGGAGAC<br>CAAGCCCTTCTGCATTGATTCCATCACCTTTGATGTGTTTGTGTGGTTT<br>GGGTGGGCTAATTCCTCCTTGAACCCCATCATTTATGCCTTTAATGCTG<br>ATTTTCGGAAGGCATTTTCAACCCTCTTAGGATGCTACAGACTTTGCCC<br>TACAA**[C/T]**GAATAATGCCATAGAGACGGTTAGCATCAATAACAATGGG<br>GCCGTGGTGTTTTCCAGCCATCACGAGCCTCGAGGCTCCATTTCCAAG<br>GACTGCAATCTGGTTTATCTGATCCCACATGCAGTGGGCTCCTCCGAG<br>GACCTCAAGAAGGAGGAGGCAGGTGGAATGGCCAGACCCTTGGAGAA<br>GCTGTCCCCGGCCTTGT	354
DRD2snp1	GGTGGTTGATTTCAGCTCTGGAGCTTTCCACGGGAGGCAGCACGCTTT<br>GGGAGGTCCTCCTACTCACTTCCTGTGTCCCCGTGTCCATTTTCCCTG<br>ACCAGAGCCTGGCCACCCAGTGGCCCCGCTGCCCCGATGGATCCACT<br>GAACCTGTCCTGGTACGATGATGATCTGGAGAGCCAGAACTGGAGCC<br>GGCCCTTCAA**[C/T]**GGGTCCGAAGGAAAGCCCGGCAAGCCCCACTACA<br>ACTACTACGCCATGCTGCTTACCCTGCTCATCTTCATCATCGTCTTCGG<br>CAATGTGCTGGTGTGCATGGCCGTGTCCCGCGAGAAGGCGCTGCAGA<br>CCACCACCAACTACCTGATTGTCAGCCTTGCTGTGGCCGACCTCCTGG<br>TGGCCACGCTCGTCATGCCCTG	23
DRD2snp2	GATGATCTGGAGAGCCAGAACTGGAGCCGGCCCTTCAACGGGTCCGA<br>AGGAAAGCCCGGCAAGCCCCACTACAACTACTACGCCATGCTGCTTAC<br>CCTGCTCATCTTCATCATCGTCTTCGGCAATGTGCTGGTGTGCATGGC<br>CGTGTCCCGCGAGAAGGCGCTGCAGACCACCACCAACTACCTGATTGT<br>CAGCCTTGC**[C/T]**GTGGCCGACCTCCTGGTGGCCACGCTCGTCATGCC<br>CTGGGTTGTCTACCTGGAGGTAGGTCTGCACCCCCGCCTGGAAGGAG<br>CTGCCCGGGGGCCTGGAGTCCAGGCTCTGTGCTGGTTCCTGCCAACA<br>AGTTGCCCGGTGCCTTCTCAGCTGGCCTGGCCTCTTCCTCCACTCTGG<br>GCTCAGTTTTTCTCCTTATAAAA	77

DRD3snp1	CACCTTTTTGATTCAATTTCCTACTCTAAAGCATAGCAGCAGGTTAGAA<br>GGTGGGTGTCAGCTGTGTGTCTCCAGTGTGGCAATACAAGCAAACACA<br>CGTACTGCCGGACACAAGGCTGGGAAGCTACAGGGTTGGGTATTTTCT<br>CCCTCTGCCCCTCCAGGGTGCTTACTTGTTGAGGGAAGCTGGGTCTGA<br>CACTGAT**[C/T]**CACTGACTGTTCTGTCGAGTGAGGATCCGTTTCCGTCT<br>CCTCTGTCTCAACACCACGTAGATCCTGGCATAGACGAGGACGGTCAC<br>TCCAAAGGGCACATAGAAGGACACCACTGAAGAGTAGATGACAAAGTC<br>AGGGTTGGAGATGGAGCACACAGTGGGGTCTCCTGTGAGTGAGAGAA<br>GAGGAGAAAGCAAAGCCAT	231
DRD3snp2	CAGGTTTTCCAAACTCGACCTAACCTAACCAAATTTAACCCAATTCATCC<br>CAGTCCAACGTGGCTCCAACTCACCAAGCACAATGGCCAGCATCTGCG<br>TTGCCTTCTTCTCCCGGAGTGGCACTCCACGAGGTTGCAAGGGCCCCA<br>GCTTCAGAGATGTCGACAACCTGCCATTACTGAGTTTTCGAACCTCTAA<br>GCTGA**[A/G]**CTTAGGTGCCATGGTGGGGCTGAGGGAGTTTCGAGTCCT<br>CCCCTCCCTTTTCAACTCCCCTCCTGCTTCTTGGAAGCCTGGCTGCCC<br>CAGGGCAGTATCTTGGCAGATGCTGTAGTAGCGCTTCAGCTCCATGTG<br>GGCCTGGCTAGGGGAGAGTGGCTGCAGTTGGGACAAGAGAGGGTCCT<br>GACATTTCTGGGGATACC	294
DRD3snp3	GCCCCAGCTTCAGAGATGTCGACAACCTGCCATTACTGAGTTTTCGAA<br>CCTCTAAGCTGAGCTTAGGTGCCATGGTGGGGCTGAGGGAGTTTCGA<br>GTCCTCCCCTCCCTTTTCAACTCCCCTCCTGCTTCTTGGAAGCCTGGCT<br>GCCCCAGGGCAGTATCTTGGCAGATGCTGTAGTAGCGCTTCAGCTCCA<br>TGTGGGCC**[C/T]**GGCTAGGGGAGAGTGGCTGCAGTTGGGACAAGAGA<br>GGGTCCTGACATTTCTGGGGATACCTTTTATCTTCTGAATGTTCCTGAA<br>AAGAAAAGAGTTTGTAAAGAAGTTCAAGGAAACGATCATTTTGTGAAAC<br>ATACTGAGGGTCACTTGAGAGTAGTGGGATCATGAAAGTTAAGGTACA<br>TGAAGTCTGCTTCTGACTT	341
DRD3snp4	TGGCCAGTGGCCCAATGGCCTGGCAGCTAAGGGTGGGCACAGGGAGT<br>ATGCTCTCCTCTCCTTCAGCAAGACAGGATCTTGAGAAAGGCTTTGCG<br>GAACTCCACATTGAAGGTGGTATAGATCACAGGGTTGAGGGCACTGTT<br>CACGTAGCCCAGCCACGTAGTGGCGCTGTAAAGCTCTGGGGACACGT<br>GGCAGGCTCC**[C/T]**CAGTGCGTATTGAGAACGTGGGTCAAAAAGAAGG<br>GCAGCCAGCAGACAATGAAGGCCCCTGGGTTGCAAAGGAAGAGAGAC<br>ATTGGTAAGGGGTTTGCTTTTCCTTTTGTTACAACATGAAAATGACTCAT<br>GGTTGTTTGCTTTGTGCCAGCCACCATGCTGGAATTTTATAGACCCATT<br>TAATTTTTCCACCAACCCTA	402
DBHsnp1	GCTTTGCCCCAGGGCGCAGGCTGGTGGGAGGGTCACCTGGACAATTG<br>AATTCCCCACCAGACAAATGTGATTAGCAGTGCAGCCTGGGCCCACCC<br>CCTGGAGGGCCAGGACATAAATGGCCCTGTGGGGTGGCCGGGCTCAC<br>GCGCAGCCATGCAGGTCCCCAGCCCCAGCGCGCGCGAGGCGGCCTC<br>CATGTACGGCAC**[A/G]**GCGGTGGCCGTCTTCCTGGTCCTCCTGGTGGC<br>CGTGCTGCAGGGCTTGGCCCCCCCCGAGAGTCCTCTCCCCTACCGAA<br>TCCCCCTGGACCCCAAGGGGGACCTGGAACTCTCCTGGGATGTCAGC<br>TACACGCAGAAGACCATCTATTTCCAGCTCCTGGTGCAAGAGCTCAAG<br>GCTGGGGTCCTGTTTGGGATGTCGGA	17
DBHsnp2	TCTCCAGACAGACTTGGAAAGTGAGAGTGATCCTCTCCAACCCCAGGA<br>AGACCCTCGGGATCCACAGAGCTGGGAGCCACACTGTGAGCAACCAG<br>CCTGGAGCTCCGGCCCTGCTTGCTTGGCCCTGGTGTTCAGGGTTGTA<br>ACCTGTCTCTGCAGGATGCCTGGAGTGACCAGCGGGGACAGATCCAC<br>CTGGATTCCCA**[A/G]**CAGGATTACCAGCTGCTGCGGGCACAGAGGACC<br>CCGAAAGGCCTATGTCTACTCTTCAAGAGGCCATTTGGCACTTGTGAC<br>CCCAAGGATTACTTCATTGAGGTGGGTGGTGGCCCTGTGTGCTCAGGA<br>GATTGTGGAGCACCCCTACCTCCTAAGGCAGCATCCCTAGGCCCCTTA<br>TGCACAGAGAGAGAGCCAGGGAG	121

DBHsnp3	CAGGGGCTGAGTAGCCGGGGATGGAATCTCAGCTGGGTCCAGGGGG<br>AACCTCAAAGGCCCCCGCCTTCACAGAAACGAGAGCCAGGCATGTCAC<br>TTGCCAGGTGAGGTGGCAGGGACCTGGCAGCGCCCGGCTACAAAGGA<br>GACCAGCTCTGGGGGGTTGGCATGTCTTGGCTGCAGGATGGCACCGT<br>CCACCTGGTGTA**[C/T]**GGGGTCTTGGAGGAGCCTTTTGGGTCGCTGGA<br>GGCCATCAACACGTCGGGGCTGCAGAAGGGACTGCAGAGGGTACAGC<br>TGCTGAAGCCCAAGATCTCCATCCCAGCCTTGCCGGAAGACAGGCGCA<br>CCATGGACATCCAAGCCCACAACGTCCTGATCCCCAGCAAGACCACGT<br>ACTGGTGTCACCTCACCAAGCTTCC	163
DBHsnp4	TCCCATTTTACAGATGGAGAAGCCGAGGCCGGGGTGGGGGGAGCCGC<br>TGGCAGAACCCCCTCCAGGGGGCGAGCAGGTGGGCGGGCGGGCATC<br>AAAGGCCCCAGGCTCAGTGCCCTCCCGCCCCCGCCCCAGTACGAGCC<br>CATCATCACCAAGGGGAACGAGGCCCTGGTCCACCACATAGAGATCTT<br>CCAGTGCACCAA**[A/C]**CAGTTCCAGAACATCACCTCATTCAGCGGGTCC<br>TGTGATTCCAAGGAGAAGCCCCAAGAGCTCAAAGTCTGCCGCCACGTG<br>CTGGCCGCCTGGGCCCTGGGTGCCAGGGTACGTGCCTTATGTCCCCC<br>CTGCAGTTTGGGGGCCCCGGCTCCTCCGCACCTGTGTCCCCTCACCT<br>CCAAGCAGGGGCCCCAAAAGGTGCT	263
DBHsnp5	AGGCTCAGTGCCCTCCCGCCCCCGCCCCAGTACGAGCCCATCATCAC<br>CAAGGGGAACGAGGCCCTGGTCCACCACATAGAGATCTTCCAGTGCAC<br>CAACCAGTTCCAGAACATCACCTCATTCAGCGGGTCCTGTGATTCCAA<br>GGAGAAGCCCCAAGAGCTCAAAGTCTGCCGCCACGTGCTGGCCGCCT<br>GGGCCCTGGG**[C/T]**GCCAGGGTACGTGCCTTATGTCCCCCCTGCAGTT<br>TGGGGGCCCCGGCTCCTCCGCACCTGTGTCCCCTCACCTCCAAGCAG<br>GGGCCCCAAAAGGTGCTTGCAAAGCCACCAGAGAGGGCCCACCCCCA<br>CCTCACTCCAACCACCACCTCACCTCACCCCCCACCTCACCCCCACCC<br>CCAGATAACCCTAAATTACATCTC	297
DBHsnp6	TCATGGGAGAAAGTAGCTGGGAAGGCAGTGCCTCGTCTCACCCCTCTG<br>GCCTCAGTTTACCTCCTGGTCCTTTCCATACAGGGTAAATGGGATCTG<br>CAACCCCTGCCTGAGATCATCTCCAAGCTAAAAGAGCCTACCCCTAGG<br>TGCCCAACCAGCCGGGATCAGAGCTCCTCCAGCCTCACTGTGGTCAAC<br>ATCGGTGG**[A/G]**GGCAAAGTTTGAGTGGGGAGCTGTCTCTCACTCCCT<br>CTTCCACGCTGTGGCTGTGGCTCACATCAGCTCCGTGCACCCCCATCC<br>TGTGAAGACCCCTGTGGAATAGCTCTGCCTGCCCAGGATGAATGGGCT<br>GGGCCGAACCCCTGCCTGAGACCATGGTCCATTCTAGCCTCCACCTCT<br>CAAGGACACCCCACCCTTGC	622
HTR1Asnp1	GGCCATCTTGCGCTTGGCCTCGGCGTTGCGCTCATTCTTCTTCTCGAA<br>GGAGGCGGGGGCGCAAGGGATGGCGCCAGCCTCGCAGGGCAGCGG<br>CAGGTGCTCTTTGGAGCTGCCCACGCGGTGCACTTCAATCACCTCCAG<br>GGCGGCGCCGTCGTCGCCCCGCCTCACCGCGCCGTTGGTGCACAGA<br>GGCCCCCCAGCCT**[A/C]**GCTCCCCGGACCCTGCCTCCATTCTCTGCCC<br>CCCGGCTCGCCGTTCACGCTCTTCCTGGGCTGCGGGGCTGGCGACAC<br>CCCGGAGCGGGCGTCCGCTCCCTTCCTCTCCGCCTTCTTGACTGTTTT<br>GCGGATGCGGAAGCGCGCGGCGCGGAAGATGCGCCCGTAGAGGACC<br>AGCATGAGCAGCAGCGGGATGTAGAAAG	270
HTR1Bsnp1	CACCTGGCCCAGCGTCCAGCGGCCGGTGACCGTGTACATGGTGCTGA<br>TGGGCATTGCCAGGATGGAGACGAGCAGGTCGGTGACGGCCAGGGA<br>GGCGATCAGGTAGTTGGCCGGCGTGTGCAGCTTCCGGGTCCGGTACA<br>CCGTGGCGATCACAAAGGCGTTGGAGAGCGTGGTGGCCAGGGTGATG<br>AGTGCCAGCAGAA**[A/C]**GACCAGGAGCACTTTCCAGGGCAGCGCGACG<br>GAGTCCTGGTAGATGTAGCCCTCGGCGCTGCAGTTGTGCGGCGCCGA<br>AGACAGGTTGGCTGGAGGAGCCCCGGTCTGGGAGCCCGCGGGCGGC<br>GGCGGGGCGCACGGAGCGCCGGCTGCTTCCATGTCTCTCCTCGCCG<br>GGGCCCCGGAGCGCAGCCCTGGGGCATGGA	53

HTR1Bsnp2	GTCCTGTAGCGGTCCAGGGCGATGACGCAGAGGTGCAGGATGGAAGC<br>CGTGCAACAGGTGATGTCCGACGACAGCCACAAGTCGCAGACCACCT<br>GGCCCAGCGTCCAGCGGCCGGTGACCGTGTACATGGTGCTGATGGGC<br>ATTGCCAGGATGGAGACGAGCAGGTCGGTGACGGCCAGGGAGGCGAT<br>CAGGTAGTTGGC**[A/G]**GGCGTGTGCAGCTTCCGGGTCCGGTACACCGT<br>GGCGATCACAAAGGCGTTGGAGAGCGTGGTGGCCAGGGTGATGAGTG<br>CCAGCAGAATGACCAGGAGCACTTTCCAGGGCAGCGCGACGGAGTCC<br>TGGTAGATGTAGCCCTCGGCGCTGCAGTTGTGCGGCGCCGAAGACAG<br>GTTGGCTGGAGGAGCCCCGGTCTGGGA	82
HTR1Dsnp1	TCCAGAGACACTCCAGGCCCTCAAGATCTCTCTCGCTCTGCTCCTCTC<br>CATCATCACGATGGCCACAGCCCTCTCGAACGCCTTTGTGCTCACCAC<br>CATCTTCCTCACCAGGAAGCTCCACACCCCGGCTAACTATCTCATTGG<br>CTCCCTGGCCATGACTGACCTCTTAGTGTCCATCTTGGTCATGCCCAT<br>CAGCATTG**[C/T]**CTATACCACCACCCGCACCTGGAGCTTTGGCCAAATC<br>CTGTGTGACATCTGGCTGTCTTCTGACATCACATGCTGCACGGCCTCC<br>ATCCTGCATCTCTGTGTCATCGCTCTGGACAGGTACTGGGCCATCACC<br>GATGCCCTGGAGTATAGTAAGCGCCGCACAGCGGGCCGGGCAGCTGT<br>CATGATCGCCACCGTCTGGG	97
HTR1Dsnp2	GCAGCTGTCATGATCGCCACCGTCTGGGTCATCTCCATCTGCATCTCC<br>ATCCCTCCGCTCTTCTGGCGGCAGGCCAAAGCTCAGGAGGAGATGTC<br>GGACTGCCAGGTGAACACATCTCAGATCTCCTACACCATCTACTCCAC<br>GTGCGGGGCCTTCTACATCCCGTCCGTGCTGCTCATCATCCTCTATGG<br>CCGCATCTA**[C/T]**GTGGCTGCCCGGAACCGCATCCTGAATCCGCCTTC<br>GCTGTACGGGAAGCGCTTCACCACAGCGCAGCTCATCACGGGCTCCG<br>CGGGGTCCTCGCTCTGCTCCCTGAGCCCCAGCCTCCAAGAGGGGCGC<br>TCGCATGCGGCCGGCCCCCCTCTCTTTTTCAACCACGTGCAAGTCAAG<br>CTGGCCGAGGGTGTCCTGGAGCG	221
HTR1Dsnp3	CTACACCATCTACTCCACGTGCGGGGCCTTCTACATCCCGTCCGTGCT<br>GCTCATCATCCTCTATGGCCGCATCTACGTGGCTGCCCGGAACCGCAT<br>CCTGAATCCGCCTTCGCTGTACGGGAAGCGCTTCACCACAGCGCAGCT<br>CATCACGGGCTCCGCGGGGTCCTCGCTCTGCTCCCTGAGCCCCAGCC<br>TCCAAGAGG**[A/G]**GCGCTCGCATGCGGCCGGCCCCCCTCTCTTTTTCA<br>ACCACGTGCAAGTCAAGCTGGCCGAGGGTGTCCTGGAGCGCAAGAGG<br>ATTTCGGCGGCCCGAGAAAGAAAAGCCACCAAAACCCTGGGGATCATC<br>CTGGGGGCCTTTATCGTCTGCTGGCTGCCCTTCTTTGTTGCATCTCTG<br>GTCCTCCCCATCTGCCGGGCCT	263
HTR1Dsnp4	ATCCTGGGGGCCTTTATCGTCTGCTGGCTGCCCTTCTTTGTTGCATCT<br>CTGGTCCTCCCCATCTGCCGGGCCTCCTGCTGGCTCCACCCAGCCCT<br>CTTTGACTTCTTCACCTGGCTAGGCTATCTCAACTCTCTCATCAACCCA<br>ATAATATACACTGTGTTTAACGAAGAGTTTCGGCAAGCGTTTCAGAGGG<br>TTGTCCA**[C/T]**GTCCGGAAAGCCTCCTAGTCTGATTTGCTGGTGACTCT<br>TGTCATCCGGTGTGTCCTGTAACCCAGCTGGAATTGTCTTGTTTCGTTT<br>TCCTGAGATTTGGGTCAATCCTGATGTCTTGGGTTTTGGTTCCATCAAT<br>AGAATTGTTCAGTGAGTGTTCTTGTTGTCTTCTTGACTTCTGGGGCACC<br>CCCTCCAAGTGGGGC	372
HTR1Esnp1	AATGAGAGGTTTGAAGTCTAAGTAGTTTCCAGCTGAGGGAAAAGAACC<br>AGCTGCCTCTCCACAGTGTAGATTGAAACAGGGTAAACATGAATCTCAC<br>TAACTGTACCACAGAAGCCAATGTGGCTGTGAGACCCAAGACCATCAC<br>TGAGAAGATGCTCATTTCCGTGACTCTGGTGATCATCACCACCCTGAC<br>CATGTTG**[C/T]**TGAACTTGGCCGTGATCATGGCCATCTGTACCACCAAG<br>AAGCTCCACCAGCCTGCCAACTACCTGATCTGTTCCCTGGCTGTGACA<br>GACCTCCTGGTGGCAGTGCTCGTCATGCCCCTGAGCATCATGTACATT<br>GTCATGGACAGCTGGAAACTAGGGTACTTCATCTGCGAGGTGTGGCTG<br>AGTGTGGACATGACCTGC	39

HTR1Fsnp1	ACTTGGCCATTCACCTCCTCCTTGGCAATCCTGCTTGCTTGTCTCTTGT<br>GGTATAATGTTTTTGCTGCTTTATATATTTTGTAGTAGAGGATCAAAATC<br>AATGTTAATGGGATGTAGAAAGCTCCAAATGTTGAGTAAATAGTGGAAA<br>CAATGTGGTCATGTTTTATGATGCACTCATCATCTCGGCTAGTTCCTTG<br>GTG**[C/T]**CTCCAGAATAAAGGAGGCATAGAAATAAAAATAGATATAATCC<br>AAACAATTATAATCATAATGCCAGCATGCTTGGGAGTCCTTTTCCTGGA<br>GTACTTCACAGCATCTGTGATCGCCCGATACCGATCCAAAGCTATAGCA<br>GAGAGATGTAAGATGGAACACGTGCAGCATGTAATGTCAACACTCAGC<br>CAAATGTCACA	162
HTR2Bsnp1	CTTTCATCACTTAATCTTTGCTAACTGGTCTGGATTACAAACAGAATCGA<br>TACCAGAGGAAATGAAACAGACTGGTGAGCAACAGGGAAAGAAACCAC<br>AGTGGGCAGCTCTTCTGATACTCACGGTGATAATACCCACAATTGGTG<br>GGAACATCCTGGTTATTCTGGCTATTTCACTGGAAAAAAAGCTGCAGTA<br>TGCTA**[C/T]**CAATTATTTTCTAATGTCCCTAGCAGTGGCTGATCTGCTAG<br>TTGGATTGTTTGTGATGCCGATTGCCCTTTTGACAATAATGTTTGGTAA<br>GTATTTCACTTTGTTCCTGATATCAAACCAGAGAACTAAAATATACTATT<br>AGAGAAATGAATACTCAGTCTTTAAAGATGGACCATTTAAAGATACAGT<br>GAGTGAACAGT	88
HTR2Bsnp2	TGTGTGGATAGGCTATGTTTCCTCAGGAGTAAATCCTTTGGTTTACACC<br>CTCTTCAATAAAACATTTCGGAATGCATTTGGCCGATATATTACCTGCAA<br>TTACCAGGCCATGAAATCAGTAAAAACTGTCAGAAAATGCTCCAGCAAT<br>AACTACTTCCGAAATGGCAGAGAACTCAAAGTTTTTCATGAAACGTGGA<br>ATG**[C/T]**GAAATGGTATTAATCCTGCCACGTACCAGAGCCCAATGAGGC<br>TCTGCAGTTCAACCATTCACGCTTCCTCAATCATTCTACTAGATACACTT<br>CTCATCACAGAAAATGAAGGTGACAAAACTGAAGAGCAAGTCAGTTATG<br>TATAGAAGGAATAGAGGTATTCACCTAATTAATATAACGATGAGTAGGG<br>TGACAAAGCA	431
HTR2Csnp1	TTCGTTCTCATTGGGTCCTTCGTCGCTTTCTTCATACCGTTGACGATCA<br>TGGTGATCACATACTGCCTGACCATCCACGTTCTTCGCCGTCAAGCCC<br>TGATGTTACTGCACGGCCACGTCGAGGAACCGCCCAGAATAAACCTGG<br>ACTTTCTGAAGTGCTGCAGGAGGAACGGCACGGAGGAAGAGAACTCT<br>GCCAACCC**[A/G]**AACCAAGATTCGAACCCACGCCGAAGAAAGAAGAAA<br>GAGCGACGTCCCAGGGGCACCATGCAAGCCATCAACAACGAACGGAA<br>AGCGTCGAAAGTCCTTGGCATTGTTTTCTTTGTGTTTCTGGTCATGTGG<br>TGCCCGTTTTTCATTACCAATATTCTGTCGGTTCTGTGCGGGAAGGCCT<br>GTAACCAAAAGCTCATGGA	280
HTR3Asnp1	TAGATTTCAAACCAGGGCAAGAGAACTGGGGGAAGCCTCGGGCTCTGA<br>TGCCCACTTTGAACTGTTCTTCCCAGCCAGGCACTGGAGGCACCTCCA<br>GGCCCAGAACACCTCCAGGCCCGCTCTGCTGAGGCTGTCAAATTACCT<br>CCTGGCCAACTACCAGAAGGGCGTGCGGCCTGTGCGGGACTGGAGGA<br>AGCCAACCA**[C/T]**GGTGTCCATCGACGTCATTGTCTATGCCATCCTCAG<br>CGTGGTGAGCACTTGACCCCAATCTGCCCAGCTCAGAGGTGGACCTTT<br>TGGGATGGGAGACCACGGAAGGCCAAGTGACCCCTGAGCAGTACAGT<br>GGACATTCAAGTACCCAGAGTACCCGTGATCCAGCACAGACCTGCGTC<br>AGGACAGACTACATCATCCAC	64
